# From carbon reduction to care quality: a stakeholder-informed agenda for decarbonizing care homes

**DOI:** 10.1093/geront/gnag162

**Published:** 2026-08-06

**Authors:** Gary Mitchell, Nuala McLaughlin-Borlace, Stephanie Craig, Tara Anderson, Daniel Hind

**Affiliations:** School of Nursing and Midwifery, Queen’s University Belfast, Belfast, Northern Ireland, United Kingdom; School of Nursing and Midwifery, Queen’s University Belfast, Belfast, Northern Ireland, United Kingdom; School of Nursing and Midwifery, Queen’s University Belfast, Belfast, Northern Ireland, United Kingdom; School of Nursing and Midwifery, Queen’s University Belfast, Belfast, Northern Ireland, United Kingdom; School of Healthcare, University of Leeds, Leeds, United Kingdom

**Keywords:** Environmental sustainability, Nursing homes, Decarbonization, Climate change, Priority setting

## Abstract

Reducing carbon emissions in care homes is not just an environmental goal; it is also a care-quality issue. Care homes have received far less attention than hospitals, despite being long-term living environments where older adults depend on buildings and organizational systems for comfort, safety, and wellbeing. Residents are especially susceptible to poor indoor environmental conditions, heat, cold, and service disruption. Drawing on the emerging literature and informed by recent stakeholder consultation with the care home sector, this Forum paper argues that decarbonization in care homes should be reframed as a gerontological care-quality priority rather than treated solely as an environmental or technical issue. The evidence base remains narrow, focused mainly on energy and emissions rather than on how low-carbon changes affect residents, staff, and the viability of care. We propose a stakeholder-informed conceptual model spanning four interrelated domains: the built environment; material and supply systems; workforce capability and culture; and governance and measurement. Reframing care home decarbonization in these terms offers a more credible basis for implementation, policy development, and future gerontological research.

## Why decarbonizing care homes now matters

Climate change is now firmly established as a major threat to human health. Its effects are already visible through rising temperatures, more frequent extreme weather events, deteriorating air quality, and disruption to the environmental conditions that sustain health and care. Older adults are especially exposed to these harms because climate-related risks intersect with multimorbidity, frailty, reduced physiological reserve, social isolation, and dependence on supportive environments and care systems (Carr et al., 2024; Prina et al., 2024; Romanello et al., 2024).

Within this wider context, long-term care requires more explicit attention than it has so far received. The World Health Organization (WHO) defines long-term care as health and social care that supports people with significant loss of physical or mental capacity to maintain functional ability, dignity, and wellbeing (World Health Organization, 2021). In the UK, care homes, including both residential and nursing homes, provide accommodation alongside personal or nursing care for people who can no longer live independently (Care Quality Commission, 2025; World Health Organization, 2019).

Questions of heating, ventilation, lighting, food, and waste are not only operational concerns, but shape everyday comfort, dignity, sleep, appetite, hydration, safety, and wellbeing. This is consistent with broader gerontological evidence that physical and social environments shape mental health and wellbeing in later life, although most of that work has focused on community and urban settings rather than institutional long-term care (Jacobs et al., 2026; Molinsky & Forsyth, 2023). In care homes, where residents spend much of their time indoors and often depend on staff and building systems to regulate their environment, indoor environmental quality is especially consequential (Gupta et al., 2021; Walker et al., 2016).

At the same time, health and care systems contribute materially to the climate crisis they must increasingly respond to. The global health sector is estimated to account for around 4.4% of net greenhouse gas emissions, and policy attention to healthcare decarbonization has grown accordingly (Karliner et al., 2019). Decarbonization refers to the process of reducing greenhouse gas emissions across systems, services, and supply chains to limit further global warming and its associated harms (Hough & Cohen Tanugi-Carresse, 2024). In health and social care, this includes emissions linked to energy use, buildings, transport, food, waste, procurement, and models of care. Its relevance is therefore twofold: decarbonization helps address the drivers of climate change while also creating opportunities for co-benefits such as cleaner air, healthier indoor environments, and more sustainable use of resources (Braithwaite et al., 2024; Jennings et al., 2020; Karliner et al., 2019). Yet most published work has concentrated on hospitals and acute care, with comparatively limited attention to long-term care settings (Braithwaite et al., 2024). That imbalance is increasingly difficult to justify. Care homes are energy-intensive, operate continuously, and serve populations with heightened susceptibility to both climate-related harm and poor indoor environmental conditions (Liu et al., 2021a, 2021b). Emerging evidence suggests that decarbonization in care homes should be understood not only in terms of carbon reduction, but also in terms of potential co-benefits for residents and organizations. A small number of studies in care homes suggest that low-carbon interventions may improve indoor environmental quality, thermal comfort, and organizational resilience, although these outcomes remain under-measured across the wider literature (Lu et al., 2019; Teni et al., 2019; Vergés et al., 2023).

This Forum paper argues that care home decarbonization should be reframed as a gerontological care-quality priority. Building on the emerging evidence base and informed by recent stakeholder engagement with the care home sector, we contend that progress will depend on moving beyond a narrow carbon-accounting perspective toward an approach that integrates resident wellbeing, workforce capability, organizational feasibility, and environmental responsibility. Existing gerontological scholarship has shown that institutional environments shape social experience, quality of life, and dependency in later life, but has not yet addressed decarbonization itself as a determinant of care quality in residential long-term care (Gruyaert et al., 2026; Liu et al., 2026; Sabharwal, 2026). This Forum paper extends that work by arguing that, in care homes, environmental change cannot be treated as a stand-alone estates or sustainability issue because it is inseparable from comfort, dependency, autonomy, and day-to-day life for older adults living with frailty, cognitive impairment, and reduced control over their surroundings.

## What the current evidence tells us and what it misses

A central limitation of the current evidence base is the relative neglect of resident-level health and wellbeing outcomes. Care home residents live within these environments continuously, and their health is directly shaped by indoor temperature, ventilation, air quality, infection risk, food quality, and the general conditions of care. Yet such outcomes are seldom measured explicitly. Anderson et al. (2025) concluded that reduced respiratory illness, improved thermal comfort, and enhanced resident wellbeing were rarely considered or quantified in care home decarbonization studies. This omission mirrors a wider problem in the health co-benefits literature. Dinh et al. (2024) found that most co-benefit studies still focus on a narrow range of health measures, particularly short-term air-pollution benefits, while neglecting broader and longer-term pathways. Jennings et al. (2020) similarly showed that the public health benefits of climate action map onto issues that matter directly to the public, including air quality, housing, food, and inequality. For care homes, this implies that the outcomes most likely to persuade staff, providers, residents, and families may be precisely those least well captured in current carbon-focused evaluation.

There are already indications of what a broader evaluative lens might reveal. WHO housing guidance links indoor temperature, accessibility, injury hazards, and housing quality to health outcomes in ways that are directly relevant to older adults with reduced physiological reserve (World Health Organization, 2018). Work on overheating in care homes has shown that the sector faces material risks from rising temperatures and that building adaptations may be justified in both health and economic terms (Gupta et al., 2021; Ibbetson et al., 2021). The limited available studies suggest that decarbonization in care homes cannot be reduced to emissions metrics alone. It must be judged in relation to the environments created for older residents and the consequences those environments have for health, comfort, and care.

A second limitation is that the existing evidence is narrow in scope, focusing predominantly on selected emissions domains rather than the full range of activities through which care homes generate environmental impact. The literature on healthcare decarbonization has expanded rapidly in recent years, but that growth has been uneven. Much of the published work has focused on hospitals and acute care, where emissions-reduction strategies are more visible, more easily measured, and more closely linked to infrastructure, clinical technologies, and procurement systems. Braithwaite et al. (2024) document a wide range of strategies to reduce the climate impact of healthcare, but long-term care does not appear. Anderson et al. (2025) identified only 22 relevant studies and found that the field is concentrated primarily around Scope 2 emissions, especially electricity monitoring and electricity reduction. Scope 1 emissions, relating to direct facility emissions, and Scope 3 emissions, relating to supply chains and wider value-chain activities, were much less developed. Food procurement emerged as one of the few comparatively developed Scope 3 areas, while broader questions around waste, pharmaceuticals, travel, and other indirect emissions remained largely absent. Organizational and behavioral dimensions of implementation were also underexplored relative to technical and infrastructural solutions.

This pattern is significant because it influences how decarbonization in care homes is conceptualized. Where evidence is concentrated on retrofit modelling, thermal systems, or electricity use, decarbonization can appear to be a primarily technical challenge. Care homes, however, are social and relational environments as much as they are buildings. Decisions about heating, ventilation, food, waste, and procurement are embedded in judgments about comfort, dignity, routines, staffing, safety, and affordability. Some of the strongest existing studies in this area already point to that complexity. Halbmayer et al. (2021), for example, emphasized the interconnections between technical and social dimensions of sustainability in Austrian nursing homes rather than treating environmental change as a purely engineering exercise. A similar point emerges in work on thermal care, where energy-related decisions are shaped by concerns about risk, responsibility, and what good care requires (Neven et al., 2015; Walker et al., 2016).

The evidence is somewhat more promising in relation to food systems. The limited Scope 3 studies in care homes consistently suggest that reducing red meat and increasing plant-based foods can lower emissions with minimal cost increases while also improving nutritional quality (Benvenuti et al., 2019; Conti et al., 2024; Lassen et al., 2021, 2025; Pörtner et al., 2025). In the limited available studies, this area may offer one of the clearest examples of how environmental and resident-centered goals can align. Yet even here the literature remains narrow, signaling opportunity rather than a settled implementation science for the sector.

## What recent stakeholder engagement adds

One reason the literature remains underdeveloped is that implementation in care homes is shaped by forms of practical knowledge that are difficult to capture through technical modelling alone. Stakeholder engagement can help by surfacing experiential, contextual, and operational perspectives that may not be visible in formal emissions studies. Broader work on involvement and engagement has shown that such processes can strengthen the relevance, acceptability, and practical usefulness of health and care research, especially in complex organizational settings (Harrison et al., 2019). In care homes specifically, effective involvement depends on recognizing the realities of the setting, including competing priorities, diverse roles, and the need for flexible and inclusive approaches (Burgher et al., 2023; National Institute for Health and Care Research, 2024).

Recent stakeholder consultation conducted in Belfast, Northern Ireland, in November 2025 further supports the relevance of this approach to care home decarbonization. The event involved more than 80 participants and was convened through an established regional nursing-home network. Participants included care home managers, nurses, care assistants, sector stakeholders, and a smaller but explicit public and patient involvement and engagement group comprising older people, caregivers of residents, and people living with conditions associated with later life. The consultation was designed to inform agenda-setting and future work in this area rather than to generate formal qualitative findings.

The event used facilitated group discussions across eight tables, supported by four facilitators. The material comprised facilitator notes, participant-completed written sheets, and plenary feedback; some elements were video-recorded with consent, although discussions were not transcribed verbatim. We therefore treat this as stakeholder-informed consultation material, reviewed to identify recurring priorities and implementation concerns.

These contributions suggested that sustainability is not experienced in care homes as an abstract net-zero objective. Rather, it is encountered through day-to-day issues such as heating control, lighting, waste segregation, food provision, procurement choices, workforce habits, financial constraints, and uncertainty about how environmental change might affect resident comfort and wellbeing. These considerations are therefore not incidental, but central to the practical viability and uptake of decarbonization in care homes.

This provides an important counterbalance to the way decarbonization is often framed in technical and policy literature. Where published studies tend to foreground what can be measured and modeled, stakeholder perspectives foreground what must be negotiated in practice, including comfort thresholds, staff routines, resident preferences, and competing operational demands. In care homes, environmental action must be negotiated within systems of care shaped by staffing pressures, aging infrastructure, constrained budgets, and the obligation to maintain comfort, dignity, and safety. The value of stakeholder consultation lies not in displacing published evidence, but in clarifying where proposed interventions are likely to gain traction, where they may encounter resistance, and which outcomes are likely to matter most to those responsible for implementation.

Four recurring priorities emerged from this consultation: the built environment; material flows, including food, waste, and procurement; workforce capability and culture; and organizational leadership, accountability, and measurement. Across these domains, participants repeatedly linked environmental change back to resident wellbeing, particularly through concerns about thermal comfort, air quality, nutrition, hydration, sleep, and meaningful activity. These priorities appear to align closely with the main gaps identified in the published literature while also sharpening their practical significance.

## Reframing decarbonization as a care-quality issue

If decarbonization in care homes is to gain traction, it must be understood not only as a contribution to net-zero targets but as a question of care quality. Three bodies of evidence support this reframing. First, the housing and public health literature has long shown that indoor temperature, ventilation, and housing quality are health issues, especially for older adults and others with reduced physiological reserve (Ormandy & Ezratty, 2012; World Health Organization, 2018). Second, a small number of studies in care homes suggest that low-carbon measures may generate co-benefits extending beyond carbon reduction, including improved air quality, better thermal comfort, and more supportive indoor environments through heat pump systems, renovation strategies, and adaptive thermal comfort approaches (Lu et al., 2019; Teni et al., 2019; Vergés et al., 2023). Third, climate and aging scholarship has drawn attention to the role of residential setting in shaping climate vulnerability and adaptive capacity in later life (Carr et al., 2024; Molinsky & Forsyth, 2023).

Recent stakeholder engagement reinforces this point by showing that care home personnel do not separate sustainability from resident experience. Concerns about heating, insulation, lighting, food, and waste were repeatedly linked to comfort, illness prevention, quality of life, and resident wellbeing. This suggests that a resident-centered framing may also be more implementable in practice. Sustainability efforts are more likely to gain traction when they are seen as supporting better living and caring environments rather than imposing external environmental goals on already pressured services and staff.

If decarbonization is to function as a care-quality agenda, it must also be understood as a workforce and leadership issue. Care home sustainability is enacted through everyday decisions and routines: how heating is managed, whether waste is separated correctly, what gets procured, how food is planned, and whether staff have the confidence and permission to change established practices. Emissions are therefore produced not only through buildings and supply chains, but also through organizational cultures and patterns of work. Recent scholarship has argued that health professionals have important roles in both climate mitigation and adaptation and that these roles need clearer definition if sustainability is to become part of normal professional responsibility rather than an optional concern (Anderson et al., 2024; Sorensen & Fried, 2024; McLaughlin-Borlace et al., 2025, 2026). Evidence on nurses’ sustainability attitudes likewise suggests that values alone are not enough; action is mediated by knowledge, confidence, and organizational context (Zoromba & El-Gazar, 2025).

Leadership is equally important. Sustainability is unlikely to become embedded unless leaders visibly frame it as organizational business, support culture change, and align environmental action with quality, ethics, and institutional mission (Sergeant & Hategan, 2023). In care homes, where decarbonization decisions often involve trade-offs around comfort, cost, staffing, and operational continuity, leadership is not simply administrative. It is interpretive. Leaders help determine whether sustainability is seen as extra work or as part of safer, healthier, and more resilient care.

Current limitations in measurement and accountability provide a further reason to frame decarbonization as a care-quality concern. Existing studies in this field rely on diverse and often highly specific forms of measurement, including organizational carbon inventory work, smart energy optimization, and building-level energy performance analysis, leaving providers and policymakers with an incomplete basis on which to judge priorities, compare interventions, or assess progress with confidence (Desmond et al., 2023; Fong et al., 2023; Kuzgunkaya, 2019).

These potential co-benefits should not be assumed to arise automatically. In care homes, low-carbon interventions may also generate trade-offs if they are poorly designed or insufficiently aligned with resident needs and organizational realities. Building changes intended to reduce emissions may compromise thermal comfort or disrupt routines if they do not account for frailty, cognitive impairment, sensory changes, or dependency (Gupta et al., 2021; Ibbetson et al., 2021; Walker et al., 2016). Dietary change may support environmental goals, but may also create tensions where changes in menus, food choice, or procurement are not matched to nutritional needs, established preferences, or the practical realities of food service in long-term care (Conti et al., 2024; Lassen et al., 2021; Pörtner et al., 2025). Additional expectations around waste segregation, procurement, or carbon reporting may also increase workload in already pressured services, particularly where staffing capacity and organizational support are limited (Davies et al., 2024; Kim et al., 2026; Mese et al., 2026; Zoromba & El-Gazar, 2025). These considerations are not arguments against decarbonization, but they do indicate that the care-quality case for action depends on explicit attention to trade-offs, implementation burden, and the possibility of unintended harm.

Carbon accounting is therefore essential, and standardized emissions reporting has been argued to be a necessary foundation for health-system decarbonization, but it is unlikely to be sufficient on its own (Singh et al., 2022). In care homes, measurement frameworks also need to capture the organizational conditions and resident-relevant outcomes that shape whether low-carbon interventions are feasible, acceptable, and sustainable in practice. This is consistent with wider implementation literature showing a gap between evidence for reducing environmental impacts and the uptake of proposed practice changes, together with limited evidence on sustained effects and barriers to innovation (Davies et al., 2024). A stronger approach would therefore connect environmental performance with outcomes that carry clear relevance in long-term care, including thermal comfort, indoor environmental quality, food quality, resident wellbeing, affordability, and organizational resilience. Framed in this way, improved carbon measurement strengthens the decarbonization agenda by making it more meaningful in gerontological practice and easier to justify within service settings. Specific outcome domains might include thermal comfort scores, respiratory infection rates, resident-reported environmental satisfaction, food quality indicators, and measures of organizational resilience.

## Toward a practical model for care home decarbonization

The literature and stakeholder-informed priorities discussed in this Forum paper indicate that progress in care home decarbonization is unlikely to come from a narrow technology-led approach alone. A more productive way forward is to understand decarbonization as a form of organizational improvement in which environmental performance, resident wellbeing, workforce practice, and service sustainability are advanced in parallel. On that basis, we propose a stakeholder-informed model of care home decarbonization that places resident wellbeing and care quality at its center and connects these to four interrelated domains of action: the built environment; material and supply systems; workforce capability and culture; and governance and measurement.

**Figure 1:**
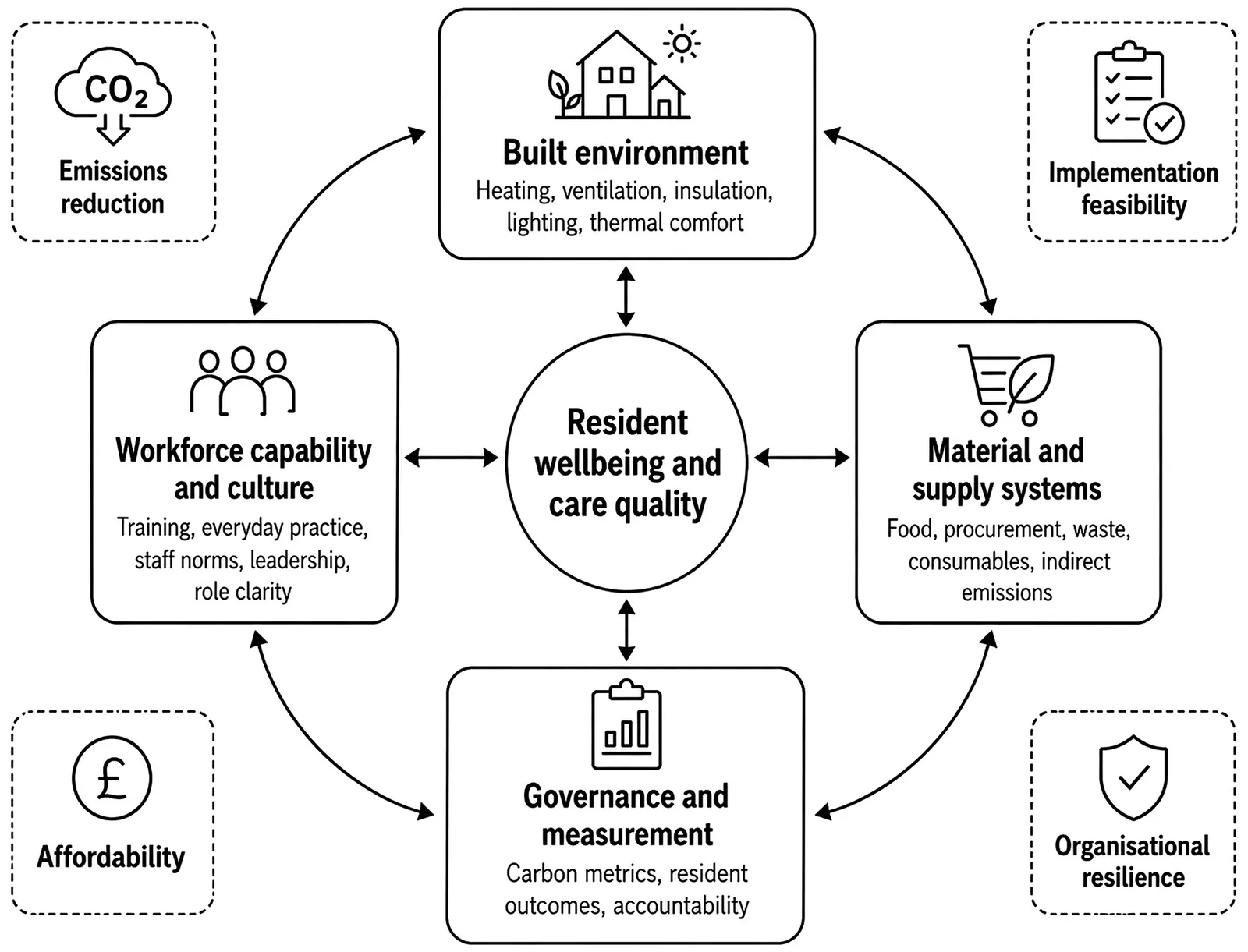
Stakeholder-informed model of care home decarbonization.

The model is intended as a guide for practice, policy, and research rather than as a validated framework. It extends broader sustainability and quality-improvement approaches by treating decarbonization as an institutional determinant of resident wellbeing and care quality in later life, rather than as a stand-alone environmental objective. Existing approaches to healthcare decarbonization often treat environmental performance as a standalone objective, assessed through carbon metrics and managed through estates or facilities functions. This model departs from that approach by positioning resident wellbeing as the organizing principle and by treating environmental, operational, workforce, and governance domains as interdependent, as illustrated in Figure 1. The model is concerned primarily with mitigation, insofar as it focuses on reducing emissions associated with buildings, energy use, procurement, food, waste, and everyday service delivery in care homes. However, some of the same interventions may also generate adaptation or resilience benefits, particularly where they reduce residents’ exposure to heat, poor indoor environmental quality, service disruption, or other climate-related risks; these wider health and organizational gains are understood here as co-benefits rather than the primary target of decarbonization.

The first domain, the built environment, includes heating, ventilation, insulation, lighting, and wider building performance. These factors are central because care homes operate continuously and because residents may be especially sensitive to thermal instability, poor air quality, and environmental discomfort. Building-related interventions are therefore likely to remain a major focus of decarbonization efforts, but their value in long-term care should be assessed not only in terms of emissions reduction but also in relation to comfort, safety, and supportive indoor environments (Gupta et al., 2021; Ibbetson et al., 2021; Walker et al., 2016). However, the effects of building change are not determined by physical infrastructure alone. They depend on how staff use new systems in practice, whether organizational leadership supports adaptation, and whether governance arrangements capture resident-relevant outcomes such as comfort and environmental satisfaction. This is especially important in care homes, where frailty, cognitive impairment, sensory changes, and dependency may heighten residents’ vulnerability to thermal stress and reduce their capacity to adapt independently to environmental change.

The second domain, material and supply systems, encompasses food, procurement, waste, consumables, and other indirect emissions pathways. The literature has only begun to address these issues, yet they are likely to be highly consequential both environmentally and operationally. Food systems are especially illustrative because they show how environmental, nutritional, and financial objectives may align (Conti et al., 2024; Lassen et al., 2021; Pörtner et al., 2025). Waste and procurement practices likewise sit at the intersection of emissions, cost, and stewardship. A practical model for the sector therefore needs to recognize that carbon reduction in care homes is not confined to buildings and utilities. It is also embedded in the routine flow of goods, services, and materials that sustain daily care. Choices in this domain are shaped by governance decisions, budgetary constraints, and staff routines, while also feeding back into resident experience through food quality, comfort, and perceptions of care (Gruyaert et al., 2026). In care homes, these choices also have distinct gerontological implications, since residents may rely on nutritionally adapted diets, continence products, assistive items, and other routine consumables that are closely tied to comfort, dignity, functioning, and everyday quality of life.

The third domain, workforce capability and culture, reflects the fact that sustainability is enacted through everyday practice. Heating is adjusted by staff, waste is sorted or mis-sorted by staff, procurement decisions are influenced by staff, and the acceptability of change is shaped through beliefs, habits, and communication. In care homes, where workloads are often intense and priorities multiple, sustainability efforts are unlikely to endure unless staff can see their relevance to resident care and receive practical support to act on them. A workable model must therefore include education, leadership at multiple levels, and attention to the cultural conditions that allow new practices to become routine. This domain also mediates whether changes in the built environment or supply systems are realized as intended, since technical or procurement interventions can be undermined or strengthened by staff knowledge, teamwork, and organizational support (Kim et al., 2026). This has salience in long-term care because residents with dementia, communication difficulties, reduced mobility, or social isolation often experience environmental change through staff interpretation, support, and day-to-day relational care rather than through infrastructure alone.

The fourth domain, governance and measurement, concerns the organizational structures through which progress is monitored, responsibilities are assigned, and priorities are reinforced. Decarbonization is difficult to embed in the absence of credible baselines, meaningful metrics, and clear mechanisms for linking environmental action to broader service goals. In care homes, governance should not be limited to reporting carbon outputs. It should also incorporate the outcomes most relevant to long-term care, including thermal comfort, food quality, resident wellbeing, affordability, and operational resilience. Therefore, this domain is not supervisory. It shapes what counts as success, influences investment and procurement decisions, and determines whether resident-relevant outcomes are recognized alongside emissions reduction. In that respect, governance and measurement connect the other three domains by defining which forms of change receive organizational attention and which outcomes are acted upon (Gruyaert et al., 2026; Kwon & Bowblis, 2026; Sabharwal, 2026). In gerontological terms, this means that governance structures help determine whether resident quality of life, autonomy, and day-to-day experience are treated as central outcomes of decarbonization or displaced by narrower operational metrics.

Collectively, these domains suggest that care home decarbonization is best understood not as a sequence of isolated interventions but as an interdependent system. Change in one area is likely to influence the others. Building upgrades may alter staff practices and resident comfort; procurement decisions may reshape waste profiles and food quality; workforce capability may determine whether technical interventions succeed in practice; and governance arrangements may amplify or constrain change across all domains. The value of this model therefore lies not only in identifying priorities, but in showing how they interact. Durable change is more likely when low-carbon action is developed and evaluated in relation to resident wellbeing, workforce capacity, and organizational resilience.

## Implications for gerontological practice, policy, and research

For gerontological practice, the central implication is that decarbonization should no longer be treated as peripheral to care delivery. In care homes, environmental conditions are inseparable from everyday residents’ experiences of comfort, sleep, hydration, nutrition, safety, and respiratory wellbeing. A more sustainable care home is therefore not simply one with a lower carbon footprint. It is one in which the physical environment, routines of care, and organizational systems are better aligned with the needs of older adults living with frailty, multimorbidity, and dependency.

At the policy level, decarbonization in long-term care requires a more developed and coherent enabling framework than currently exists. National and regional sustainability strategies have tended to privilege hospitals and acute care, leaving care homes comparatively under-specified within health-system decarbonization agendas. That imbalance is difficult to justify given the scale, intensity, and vulnerability profile of the sector. A more mature policy response would support care homes through clearer standards, proportionate expectations, accessible guidance, and investment frameworks capable of addressing the distinctive constraints of long-term care settings. One practical step would be the incorporation of environmental sustainability into regulatory inspection frameworks. Capital funding streams for building retrofit should also be extended explicitly to the care home sector, which has historically been excluded from infrastructure investment programs designed for acute care. Sector bodies, regulators, commissioners, and funders each have a role in enabling this agenda, including through the development of clearer standards, accessible guidance, proportionate inspection approaches, and investment mechanisms that recognize the practical constraints of care-home settings.

For organizational leadership, the implications are similarly clear. Sustainability in care homes will not be secured by technical solutions alone. It requires visible leadership, practical training, role clarity, and forms of accountability that connect environmental goals with service priorities. A care-quality framing offers one route through the tensions that often accompany environmental action by placing decarbonization alongside, rather than in competition with, the core purposes of care. Within organizations, care home managers and senior providers are central to this process through their influence on staff training, role allocation, procurement decisions, and the prioritization of sustainability alongside resident care and operational demands.

The implications for research are perhaps the most immediate. Current scholarship has mapped important aspects of the field, but it has not yet produced a sufficiently integrated understanding of what works, in which contexts, and with what consequences for residents, staff, and providers. Three priorities follow. First, future studies should extend beyond narrow carbon accounting to include outcomes of direct relevance to long-term care, such as thermal comfort, indoor environmental quality, food quality, resident wellbeing, and service resilience. Second, implementation research is needed to examine how interventions are adopted, adapted, sustained, or resisted within real-world care home settings. Third, greater attention should be given to supply chains, procurement, waste, and other indirect emissions sources that remain weakly developed in the literature but are likely to be highly consequential. Researchers should also work with residents, relatives, care staff, managers, and sector stakeholders to co-develop studies that examine resident-relevant outcomes, implementation processes, and the organizational realities of procurement, waste, and supply-chain change. Cross-national comparative research is needed to establish whether the patterns identified here, particularly the concentration on energy use and the neglect of resident-level outcomes, hold across different regulatory, funding, and organizational contexts, or whether some systems have progressed further than others. The emerging long-term care sustainability literature also suggests that environmental sustainability should be placed alongside economic, social, and technological dimensions rather than treated in isolation (Park et al., 2025).

These implications point toward a broader reorientation in gerontological scholarship. Decarbonization should not be viewed solely as a technical, estates, or environmental issue delegated to others. In care homes, it belongs within wider goals of creating healthier, safer, and more sustainable environments for later life.

## Conclusion

Care home decarbonization has begun to attract scholarly and policy attention, but the field remains at an early stage of development. The available literature shows an emerging but fragmented evidence base, with much of the work concentrated on energy use and relatively little attention paid to supply chains, organizational implementation, or outcomes that matter directly to residents. At the same time, stakeholder engagement indicates that the sector is already grappling with many of the practical questions that the literature has only partially addressed, including how to balance environmental goals with comfort, dignity, affordability, staff capacity, and the everyday realities of long-term care.

This Forum paper has argued that progress will remain limited unless decarbonization in care homes is reframed as a gerontological care-quality issue. Such a reframing places resident wellbeing, workforce capability, organizational feasibility, and environmental responsibility within the same field of concern. It also helps to make conceptual sense of care homes as places where older adults live as well as receive care. In these settings, emissions reduction cannot be judged solely through technical or managerial indicators. It must also be considered in relation to the environments created for residents and the conditions under which staff provide care.

A practical way forward lies in treating care home decarbonization as an integrative agenda spanning the built environment, supply systems, workforce culture, and organizational governance. The stakeholder-informed model proposed here (Figure 1) is intended as an initial contribution to that task. It does not resolve the significant empirical and policy gaps that remain, nor does it substitute for the need for future research. It does, however, offer a conceptual structure through which decarbonization can be discussed in terms more closely aligned with gerontological practice and long-term care. If the sector is to move beyond broad endorsement of sustainability and toward credible forms of change, the next phase of work will need to connect emissions reduction more firmly to the quality, safety, and lived experience of care.

## Data Availability

The authors do not report data, and therefore the pre-registration and data availability requirements are not applicable.
